# Supplementary material for: The C Terminus of the Ribosomal-Associated Protein LrtA Is an Intrinsically Disordered Oligomer
Source: Int J Mol Sci. 2018 Dec 5;19(12):3902. doi: 10.3390/ijms19123902 (PMC6321479; doi:10.3390/ijms19123902)

The C terminus of the ribosomal-associated protein LrtA is an intrinsically disordered oligomer

José L. Neira, A. Marcela Giudici, Felipe Hornos, Arantxa Arbe and Bruno Rizzuti

SUPPLEMENTARY FIGURE LEGENDS

FIGURE S1: **Far-UV CD spectra of C-LrtA.** Raw spectra of C-LrtA at two different concentrations; the spectrum acquired at the highest concentration (20 μM, in protomer units) was normalized to that at the lowest concentration (10 μM, in protomer units).

FIGURE S2: **Size exclusion chromatograms of C-LrtA.** Chromatograms obtained at 500 μM (black) and blue 30 μM (blue) (in protomer units) of C-LrtA in an analytical Superose 12 10/300 GL column, at pH 8.0 (50 mM Tris) and 0.250 M NaCl. The arrows from left to right indicate the elution volumes of: blue dextran (7.34 mL); ferritin (10.11 mL); catalase (11.24 mL); albumin (12.26 mL); RNase A (15.08 mL); and the bed volume, as measured by conductivity of the solution (19.15 mL). The intensity of the chromatogram for the most diluted protein concentration was increased fifteen-times to allow for a comparison.

FIGURE S3: **Purification and self-association of C-LrtA.** (A) SDS-PAGE of C-LrtA purification. The marker used was PAGEmark Tricolor (lane (3)), with markers (from top to bottom) of 210, 110, 67, 48, 32, 10, 16 and 6 kDa. Lane (2): whole LrtA (molecular weight 22.7 kDa). Lane (1): pure C-LrtA after the Hi-Trap Mono Q step (molecular weight of 12.5 kDa). Bis-acrylamide concentration of the gel was 12%. (B) BN-PAGE of C-LrtA at different SDS concentrations; the sodium channel protein from *Magnetococcus marinus* is shown as a comparison, the molecular marker is shown at the side. The arrows indicate the self-associated species of C-LrtA described in the main text. (C)

SDS-PAGE of C-LrtA at different times after addition of glutaraldehyde cross-linker. Lanes (1) and (2): PAGEmark Tricolor; lane (3): C-LrtA after 1 min of cross-linker addition; lane (4): C-LrtA after 30 minutes of cross-linker addition; and lane (5): C-LrtA after 15 minutes of cross-linker addition. The bis-acrylamide concentration of the gel was 12%.

FIGURE S4: **ITC thermogram**: Heat evolved upon dilution of C-LrtA into the calorimetric cell containing water: raw data (up) and processed data (bottom). The concentration of the protein in the syringe was 498 μM.

Fig. S1 (Neira et al.)

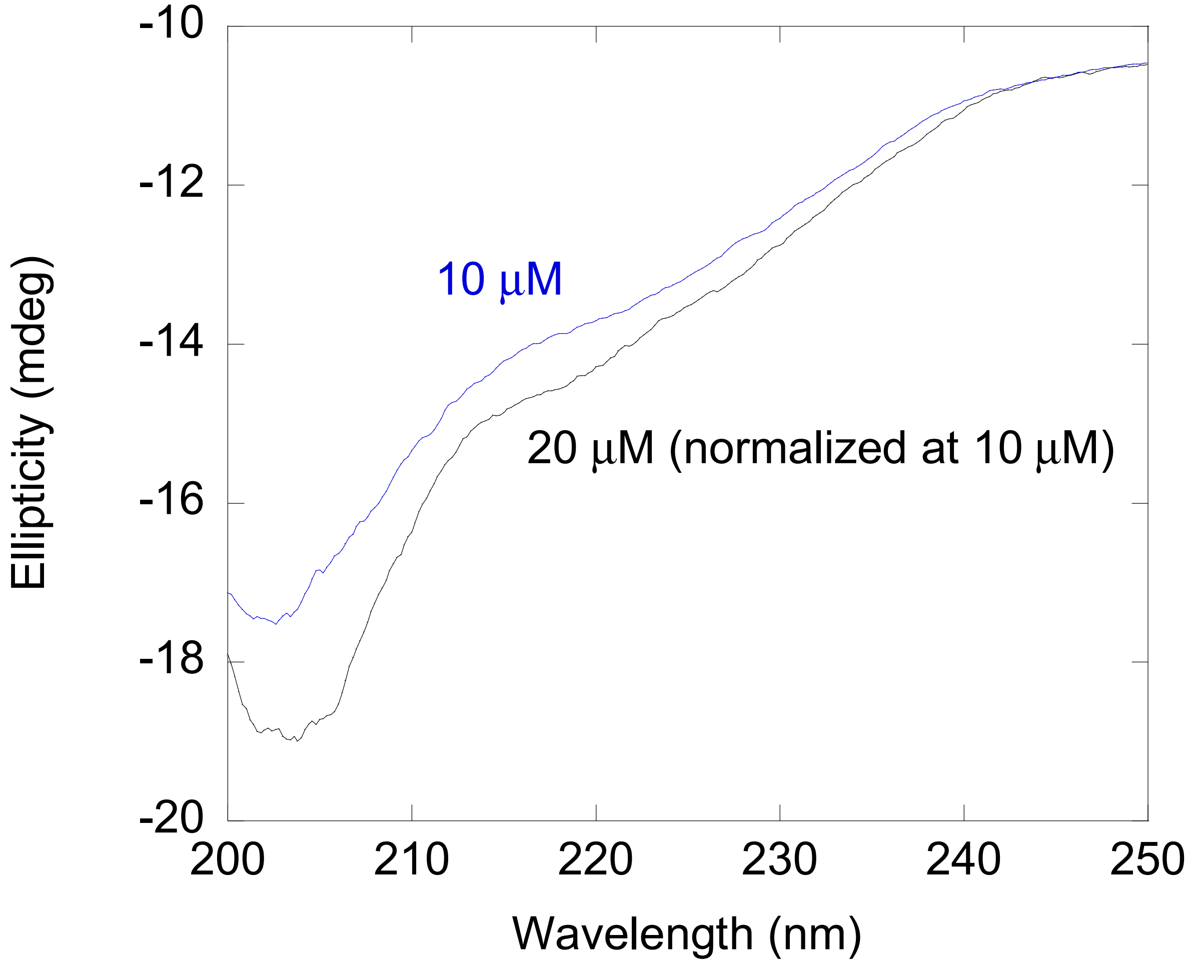

Fig. S2 (Neira et al.)

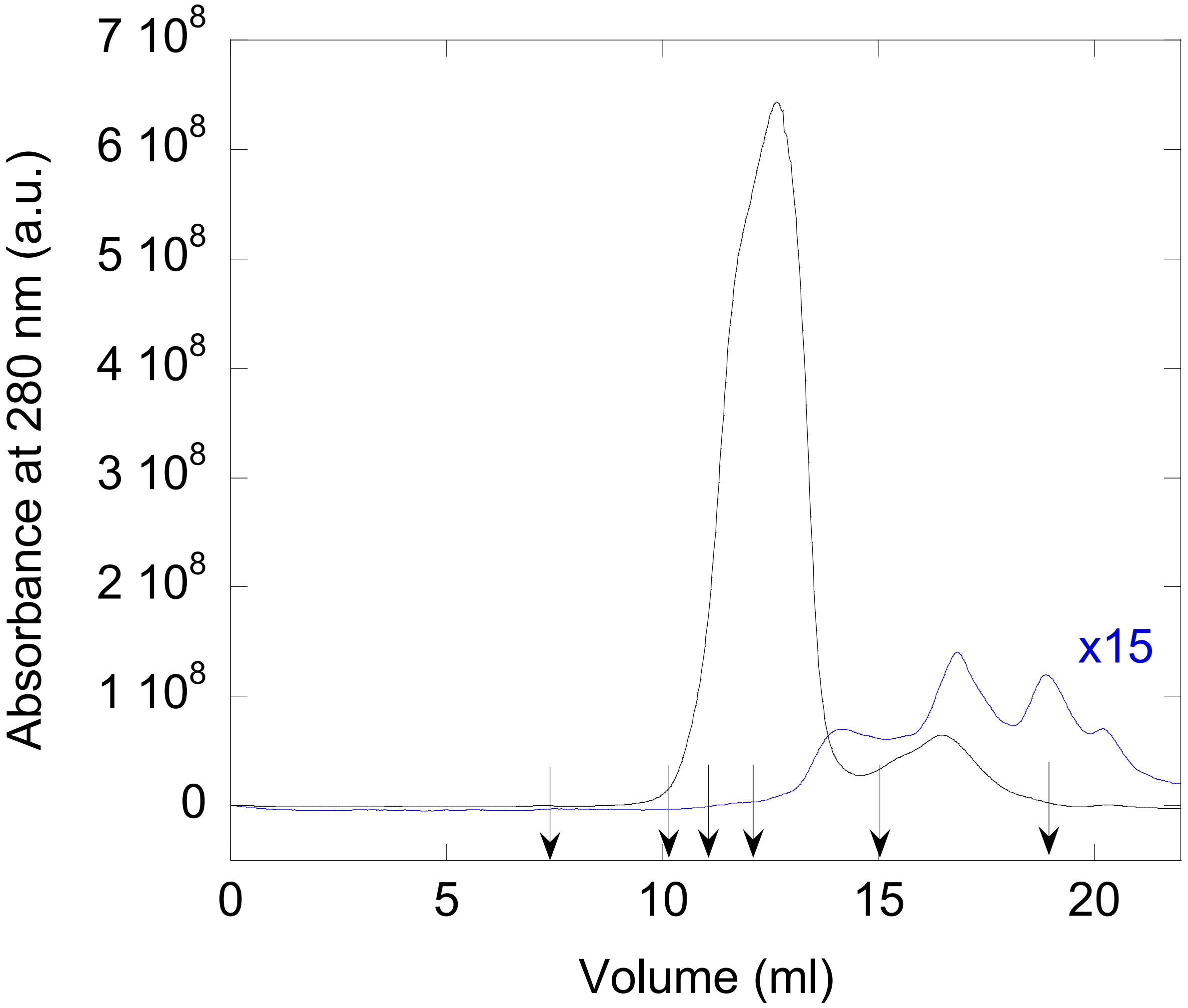

Fig. S3 (Neira et al.)

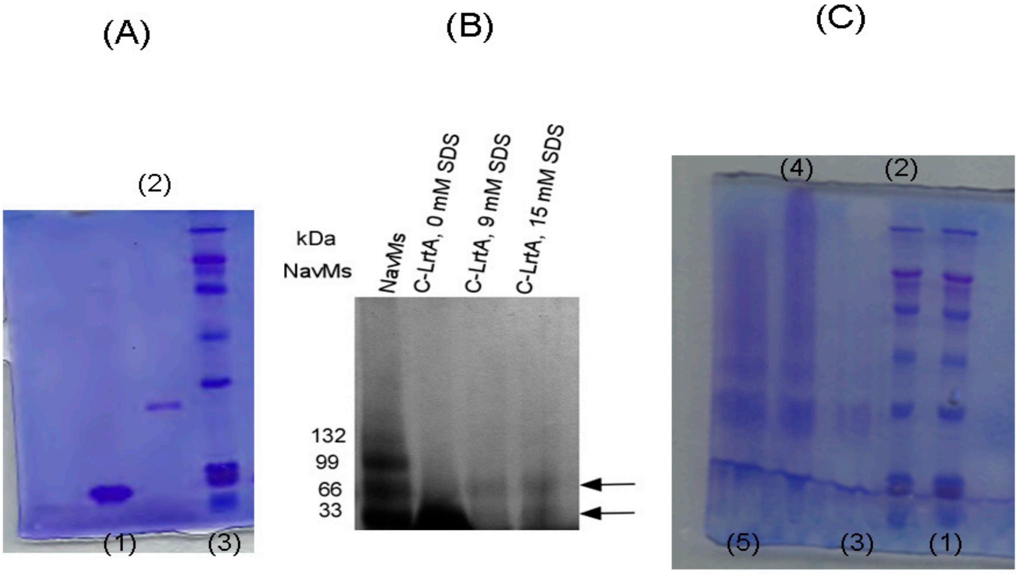

Fig. S4 (Neira et al.)

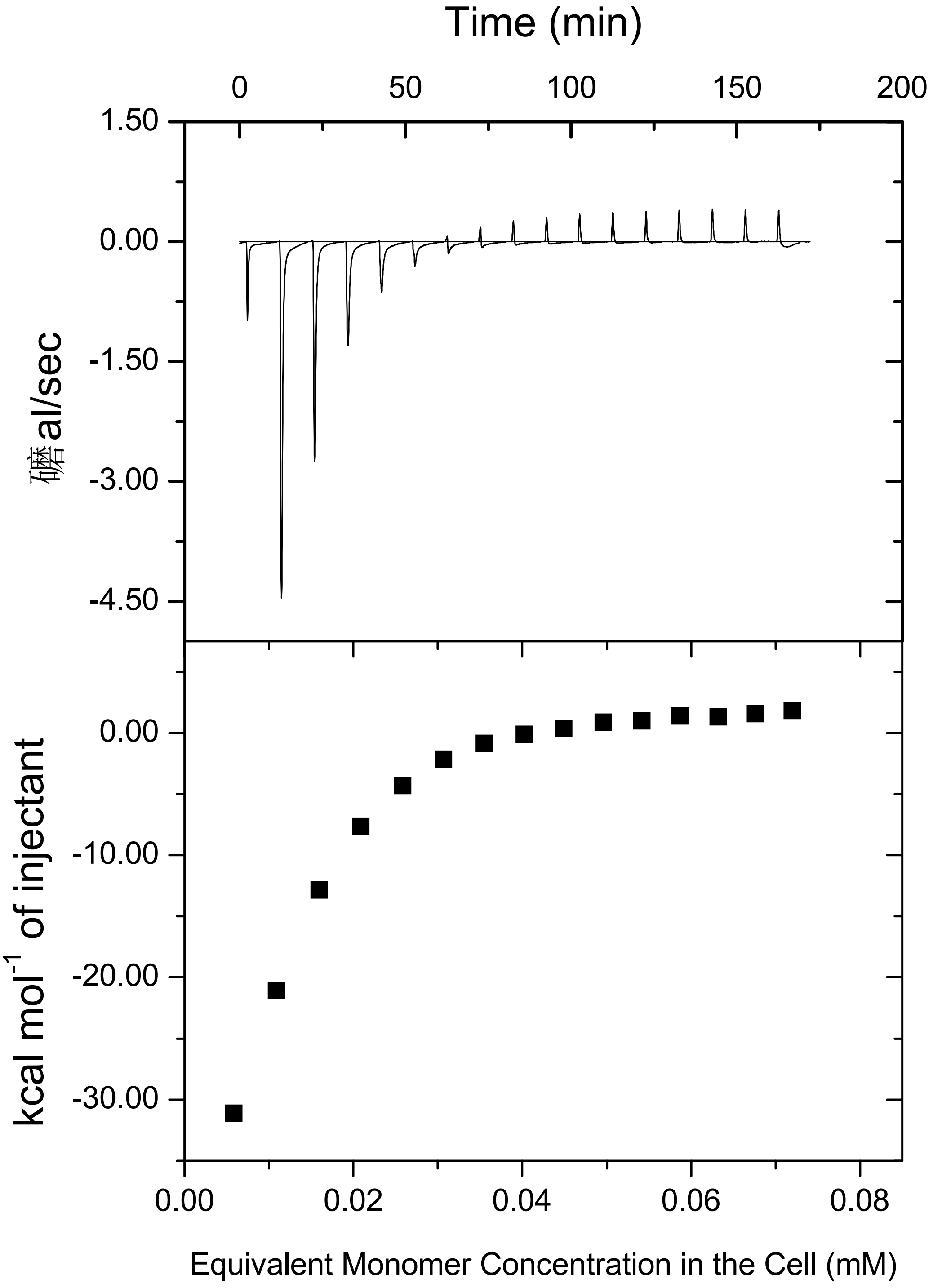

Supplement: Supplementary file 1 [file ijms-19-03902-s001.pdf]
